# The Postprandial Appearance of Features of Cardiometabolic Risk: Acute Induction and Prevention by Nutrients and Other Dietary Substances

**DOI:** 10.3390/nu11091963

**Published:** 2019-08-21

**Authors:** Laurianne Dimina, François Mariotti

**Affiliations:** UMR PNCA, AgroParisTech, INRA, Université Paris-Saclay, 75005 Paris, France

**Keywords:** metabolic syndrome, postprandial, endothelial function, oxidative stress, nuts, berries

## Abstract

The purpose of this review is to provide an overview of diets, food, and food components that affect postprandial inflammation, endothelial function, and oxidative stress, which are related to cardiometabolic risk. A high-energy meal, rich in saturated fat and sugars, induces the transient appearance of a series of metabolic, signaling and physiological dysregulations or dysfunctions, including oxidative stress, low-grade inflammation, and endothelial dysfunction, which are directly related to the amplitude of postprandial plasma triglycerides and glucose. Low-grade inflammation and endothelial dysfunction are also known to cluster together with insulin resistance, a third risk factor for cardiovascular diseases (CVD) and type-II diabetes, thus making a considerable contribution to cardiometabolic risk. Because of the marked relevance of the postprandial model to nutritional pathophysiology, many studies have investigated whether adding various nutrients and other substances to such a challenge meal might mitigate the onset of these adverse effects. Some foods (e.g., nuts, berries, and citrus), nutrients (e.g., l-arginine), and other substances (various polyphenols) have been widely studied. Reports of favorable effects in the postprandial state have concerned plasma markers for systemic or vascular pro-inflammatory conditions, the activation of inflammatory pathways in plasma monocytes, vascular endothelial function (mostly assessed using physiological criteria), and postprandial oxidative stress. Although the literature is fragmented, this topic warrants further study using multiple endpoints and markers to investigate whether the interesting candidates identified might prevent or limit the postprandial appearance of critical features of cardiometabolic risk.

## 1. Introduction

This review focuses on the kinds of diets, food, and food components that affect postprandial inflammation, endothelial function, and oxidative stress, and which are related to cardiometabolic risk, including metabolic syndrome (MS), and ultimately, cardiovascular diseases (CVD) and type 2 diabetes. Although this review gathered a very large number of studies, it is not intended to be exhaustive; rather, it emphasizes the food and food components that have been studied the most, and the data that together help us to understand the impact of nutrition on cardiometabolic risk, as this can be studied during the postprandial period. 

Metabolic syndrome refers to the clustering of a series of risk factors for CVD, whose prevalence is rising markedly at a global level [1,2,3,4,5]. Because MS is an important risk for CVD and type-II diabetes [6], considerable attention has been paid to analyzing its links with environmental factors and diet.

MS has been characterized from a clinical point of view using the following criteria: a high waist circumference; raised plasma triglycerides, plasma glucose, and systolic blood pressure; and lower HDL-cholesterol concentration [5,7,8]. From a pathophysiological viewpoint, the heterogeneity of MS is considerable, but there is now consensus regarding the importance of a few related features that are major components of cardiometabolic risk. MS is mainly considered as being related to the development of resistance to the action of insulin in different tissues and on different metabolisms [9], linked closely to the onset of systemic low-grade inflammation, which in turn is associated with the development of abdominal fat [10]. The third element in the triad is the initiation of vascular endothelial dysfunction. Indeed, endothelial dysfunction is closely associated with insulin resistance and it is the manifestation of a pro-inflammatory and pro-atherogenic phenotype in the vascular milieu [8,11]. Nutrition, and in particular western diets, have been implicated in the onset of this cardiometabolic risk; for a review see [12,13,14]. Controlled studies in animals have provided further evidence that insulin resistance, systemic and adipose tissue low-grade inflammation, and vascular endothelial dysfunction, as promoted by western diets, are early features of this cardiometabolic risk cluster [15].

From a mechanistic standpoint, a growing body of evidence is tending to confirm the rationale for a close association between insulin resistance and endothelial function. Firstly, it has been suggested that endothelial dysfunction is the earliest manifestation of diet-induced cardiometabolic risk, even before the onset of insulin resistance and a systemic inflammatory state [15,16,17,18]. Secondly, endothelial dysfunction may be largely driven by an impairment of the action of insulin on the endothelium, so that this dysfunction could be considered as a vascular feature of insulin resistance, itself promoting a pro-inflammatory state in the vascular milieu [19,20]. In turn, macro- and micro- vascular endothelial dysfunction limits the action of insulin on the peripheral extraction of nutrients by limiting the perfusion of insulin-sensitive tissues [21,22]. Endothelial dysfunction and insulin resistance would thus interact in a reciprocal relationship [20,23,24,25]. Abnormal nitric oxide (NO) production or signaling and endothelial dysfunction, triggered by excessive exposure to high-fat and high-sucrose foods, may be one important mediator of diet-induced insulin resistance and cardiometabolic risk [26,27].

## 2. The Postprandial Period as a Metabolic Challenge Eliciting Pathophysiological Features Related to Cardiometabolic Risk

A very large body of evidence has demonstrated that a metabolic challenge with a high saturated fat and high sucrose meal results in the transient appearance of low-grade inflammation and endothelial dysfunction [28,29,30,31,32,33,34,35,36,37,38,39,40].

The level and chronology of these phenomena are closely associated with the postprandial rise in plasma glucose and lipids [35,41,42,43,44]. Postprandial inflammation has been characterized at a systemic level [38,45], in blood leukocytes [42,46,47], in the visceral adipose tissue [48,49], and at the vascular level as an increase in intercellular or vascular adhesion molecules and proteins measured in the plasma ICAM-1 et VCAM-1 [32,50]. Other postprandial changes associated with inflammation have been reported after a high fat meal (HFM), such as changes to markers of angiogenesis (vascular endothelial growth factor-VEGF) [51]. Postprandial vascular endothelial dysfunction has also been repeatedly documented using integrative physiological endpoints such as macrovascular reactivity to acute changes in shear stress (particularly using flow-mediated dilation of the brachial artery-FMD) [52,53].

Although the underlying mechanisms are not fully elucidated, the dramatic rise in plasma glucose and triglycerides (and more precisely chylomicrons and their remnants) are considered to be the trigger factors for the activation of inflammatory signaling pathways in leukocytes, endothelial cells, and possibly other cells or tissues [35,48,54,55,56]. Postprandial oxidative stress is one mediator of the effect of metabolic stress on inflammation and vascular dysfunction [57,58]. Early evidence for the contribution of oxidative stress was provided by the finding that pre-treatment with high doses of vitamin C and/or vitamin E blunted postprandial endothelial dysfunction and inflammation [32,59]. As we also discuss further below, the initiation of low-grade endotoxemia is considered to be an important mechanism [47,60]. Lastly, of importance to our understanding of cardiometabolic pathophysiology is the fact that postprandial inflammation and macro/micro- vascular endothelial dysfunction are all the more important if individuals present at baseline with markers of dysregulation or cardiometabolic risk factors [21,61], and dysfunction increases when the meal challenge is repeated [62].

At the molecular level, considerable importance has been given to NO, primarily because it is well-known as the pivotal molecule of vascular health, and endothelial dysfunction can be explained by alterations to NO synthesis and/or bioactivity. More specifically, regarding postprandial deregulation the role of NO in the insulin-mediated peripheral extraction of nutrients is becoming increasingly well-established [19,22,63,64,65,66,67,68]. Furthermore, high fat and high sucrose meals impact NO synthesis and/or NO downstream signaling [26,69,70], and studies have confirmed that impairment of the insulin sensitivity of the vascular NO production pathway may explain the impairment of glucose extraction in the muscle [20,23,24,71]. Finally, because the NO pathway is more sensitive to the oxidative/redox state at many different levels, this pathway may mediate the effect of a postprandial increase in oxidative stress on impairment of endothelial function and the initiation of vascular and systemic inflammation.

The final picture is that the postprandial occurrence of low-grade inflammation and endothelial dysfunction is extremely relevant to the pathophysiological influence of nutrition on cardiometabolic risk for the following reasons: (i) low-grade inflammation and endothelial dysfunction are well known to be pivotal to the initiation and progression of cardiometabolic dysregulations, as discussed previously; (ii) their postprandial appearance is directly related to the degree to which energy nutrients challenge homeostasis and are concurrent with deregulations at the cellular and molecular levels; (iii) their postprandial appearance is graduated according to the basal level of metabolic regulation and in line with the existence of risk factors for CVD and type-II diabetes; and (iv), the level of the postprandial rise in plasma triglycerides, and glucose after a meal challenge is considered to be a potent risk factor for CVD and type-II diabetes [72,73,74]. Finally, the current paradigm is that repetition of these adverse, silent postprandial events is a mechanism for the initiation and progression of metabolic dysregulation, CVD, and type-II diabetes [36,73].

Accordingly, the postprandial state following a challenge meal offers an interesting, practical, and relevant model for studying the impact of nutrients on metabolic dysregulation, and the initiation of cardiometabolic risk factors such as MS.

## 3. Fatty Acids, Carbohydrates, and Postprandial Adverse Effects

As mentioned above, there is very convincing evidence that a challenge meal containing both saturated fatty acids and sucrose triggers a vast corpus of inflammatory phenomena and endothelial dysfunction features during the postprandial period. A smaller, yet still high, number of studies have also reported similar findings when the challenge meal only contained saturated fat or simple sugars [75,76,77,78], although some studies using a single macronutrient were negative [75]. It should be noted that these studies differed markedly in terms of the methods used to study postprandial metabolism [74].

### 3.1. Fatty Acids in Challenge Meals

In contrast, the literature is less conclusive regarding the role of the type of fatty acids in the challenge meal [79]. It should, however, be noted that olive oil (as compared to oils rich in palmitic acid, or to milk fat) induces a smaller increase in plasma inflammatory markers, does not result in activation of the NF-κB inflammatory pathway in peripheral blood mononuclear cells, and generates less postprandial endothelial dysfunction in healthy individuals and/or those with risk factors [80,81,82].

When supplementing a high fat meal, fish oils have also been shown to be beneficial to postprandial vascular function. In a postprandial model combining a high-fat meal and a heparin infusion to increase postprandial non-esterified fatty acids (NEFA), the standard high-fat meal with saturated fatty acids (SFA) impaired flow-mediated dilation (FMD) whereas the addition of fish oil to this meal conversely improved FMD 4 h after ingestion [83]. In another study, the introduction of fish oil as part of a high-fat meal improved (endothelium-independent) microvascular reactivity and increased postprandial plasma nitrite concentration (a marker of nitric oxide synthase activity) [84]. Fish oil enhanced eNOS expression in cultured endothelial cells exposed to triglyceride-rich lipoprotein isolated after the meal. When associated with fibers, unsaturated fatty acids (unSFA) blunted the postprandial expression of the inflammatory genes usually found after a high SFA meal; that is, the postprandial circulation levels of IL-1β, IL-6, MCP-1, and IFN-γ did not rise after an unSFA and fiber-rich meal when compared with an SFA meal [85].

An antioxidant and anti-inflammatory effect of olive oil or monounsaturated fatty acids (versus saturated fatty acids and low-fat meals) during the postprandial state has also been reported when the individuals had been receiving diets of a similar composition before the postprandial challenge [86,87].

The underlying mechanism for the effect of SFA on systemic inflammation has been documented. Studies have suggested that SFA increase the intestinal absorption of lipopolysaccharide (LPS), which in turn increases postprandial endotoxemia and the postprandial inflammatory response. For instance, in individuals with metabolic syndrome, a meal rich in SFA raises plasma LPS concentrations when compared to other meals rich in monounsaturated fatty acids (MUFA) or low in fat, and high in complex carbohydrates and n-3 fatty acids. After the SFA meal, the increase in LPS was correlated with the gene expression of IkBα (an NF-kB inhibitor) and MIF1 (a pro-inflammatory cytokine) in peripheral blood mononuclear cells, suggesting partial mediation by these pro-inflammatory pathways [88,89]. Finally, a high SFA meal could be involved in causing postprandial endotoxemia and also affect other mechanisms, including intestinal absorption and clearance rates of LPS, changes to intestinal microbiota, and intestinal barrier function [88]. However, it remains difficult to assess the significance of endotoxins in plasma, as LPSs are highly heterogeneous. Indeed, stimulatory, non-stimulatory, and inhibitory LPS molecules coexist in plasma, and assays cannot distinguish or quantify them separately [90].

In contrast, the literature remains scarce and still inconclusive regarding the effect of different types of saturated fatty acids, or the role of various unsaturated fatty acids [91,92,93,94,95].

### 3.2. Carbohydrates in Challenge Meals

There is quite a large body of evidence to suggest that sucrose and glucose loads induce postprandial inflammation and endothelial dysfunction, related to the postprandial increase in plasma glucose [75,96], although there have been some negative reports when these loads were given alone (i.e., without saturated fatty acids). To our knowledge, there are no data regarding the effect of other simple carbohydrates. Given the relationship between postprandial plasma glucose and postprandial dysfunctions, the glycemic index (GI) is expected to be an important factor in the adverse effect of carbohydrates, however, findings are scarce and conflicting [56,97,98]. For instance, nuts have shown potential to manage post-meal glucose when consumed with high GI food content [99] but not with low GI foods [100]. Also, the acute ingestion of low-fat milk has been shown to protect adults with metabolic syndrome from endothelial dysfunction when compared to rice milk (high GI). The postprandial serum glucose peak was higher after rice milk and correlated positively with an increase in malondialdehyde (MDA, a biomarker of oxidative stress mostly related to lipid peroxidation) and a drop in plasma arginine, suggesting that cow’s milk may limit postprandial hyperglycemia, which in turn may decrease lipid peroxidation and enhance NO bioavailability [101].

Although most studies have resorted to using experimental artificial meals containing high amounts of simple ingredients such as milk cream and sucrose, postprandial inflammation and dysfunction are not the result of an experimental artefact because they have also been evidenced following the consumption of “real” energy-dense meals, such as those supplied by fast-food outlets [46,102,103,104,105]. In contrast, some foods, such as orange juice and certain meals considered to form part of a prudent diet (e.g., meals rich in fibers and fruit, or light regular meals), do not induce adverse postprandial effects [106,107,108,109,110].

## 4. Relevance to the Effect of the Type of Dietary Protein

As mentioned before, some carbohydrates and fat sources do not appear to elicit any adverse effects during the postprandial period. Although dietary proteins are the third most important energy macronutrient, their effects have been little studied.

Indeed, we previously reported that a mixture of 50 g amino acids (based on the total milk protein composition, and with or without a supplement of l-arginine) did not increase plasma markers of inflammation or induce endothelial dysfunction [111].

In a pioneering work, Westphal and colleagues showed that adding dietary protein (milk or soy protein) to a high-fat meal prevented postprandial endothelial dysfunction [112]. This effect could, however, be explained by a quantitative effect of protein, because a high intake of protein (as compared to fat), (i) slowed down gastric emptying and decreased postprandial exposure to fatty acids in the meal [113], and (ii), raised postprandial insulin, which in this context could have anti-inflammatory and anti-atherogenic properties [114]. However, specific effects of protein quality or specific amino acids have also been documented [115]. The same authors reported that a “dietary” amount (2.5 g) of l-arginine alone (and not phenylalanine or leucine) prevented postprandial endothelial dysfunction [78], confirming the results of a study that used a massive dose of l-arginine [116]. The issue of the dose was raised in one of our studies which consisted of supplementing overweight adults with a low dose of l-arginine. After a high fat meal, reductions in the FMD and fRHI (a reactive hyperemia index that is another measure of endothelial function) compared to baseline were attenuated by arginine supplementation in individuals whose plasma arginine concentration was below the median [117]. Likewise, in a validated rat model [70], we showed that rapeseed protein (an arginine- and cysteine- rich protein when compared to milk protein), and the supplementation of milk protein with l-arginine and l-cysteine, prevented postprandial endothelial dysfunction [118]. Using this model, we were also able to show that rapeseed protein markedly reduced a postprandial increase in the production of reactive oxygen species (ROS) in the aorta [70]. Indeed, dietary arginine and cysteine are known to impact critical metabolic pathways (notably glutathione and nitric oxide) and may exert favorable effects on the initiation of cardiometabolic risk factors such as insulin sensitivity and endothelial function [119,120].

It has also been reported in overweight/obese individuals that neither a palmolein nor an olive oil diet impaired postprandial FMD when consumed in a high-fat, high-protein meal rich in l-arginine [121]. These results were not in line with the findings of a study that could not find a protective effect of proteins on postprandial endothelial dysfunction and low-grade inflammation, apart from a decrease in sVCAM after a protein mix compared to maltodextrin. However, the protein mix that was used during that study was not high in arginine, and this might have been the reason for the discrepancy [122].

Other plausible mechanisms (other than the arginine content) could explain the protective effect of milk on cardiometabolic health and endothelial function [123,124,125]. For example, acute dairy cheese consumption has been demonstrated to improve NO-dependent vasodilation compared to non-dairy products (soy cheese and pretzels) when eaten with non-dairy sodium. This suggests that dairy proteins may protect against Na-induced reductions in NO-dependent dilation [126].

## 5. Foods, Nutrients, and Other Dietary Substances That May Protect against Adverse Postprandial Effects

The adverse postprandial effects of a high-saturated fat/high-sucrose meal have been used to determine whether adding a nutrient or dietary substance to that meal might lower or prevent the postprandial inflammatory reaction and endothelial dysfunction. Because high exposure to triglycerides and glucose have been convincingly proposed as trigger factors for adverse postprandial effects, numerous studies have addressed the effects of dietary factors on postprandial increases in glucose and triglycerides. As with the addition of protein, some foods or ingredients may basically act through their added weight/energy, slowing down gastric emptying and modulating plasma insulin. Furthermore, the kinetics of digestion and the availability of carbohydrates and fats differ depending on the type of food or the structure of the meal. For instance, the unique physical structure of nuts may explain their role in postprandial regulation. Indeed, the effects of processing on nuts have been shown to affect the postprandial glycemic response [127] by breaking down the nut cell walls and increasing the bioaccessibility of intracellular lipids [128,129], leading to prolonged gastric emptying. Likewise, we have shown that interactions between macronutrients within a meal may modify the kinetics of the absorption of meal fat and result in a different challenge for postprandial metabolism [130,131].

Several nutrients, micronutrients, and phytochemicals may affect postprandial blood lipid concentrations after both acute and chronic consumption, as recently reviewed in detail by Desmarchelier et al. [132]. Among many examples [133], a blend of antioxidant spices added to a high-fat meal lowered postprandial insulin and triglycerides [134]. Nuts have also been described as improving postprandial FMD [135,136], glycemia [137,138], and triglyceridemia [139]. In contrast, in many cases, certain nutrients and other dietary substances that have been shown to reduce the adverse postprandial effects of a challenge meal, did not affect postprandial plasma lipids [140].

### 5.1. Adding Nuts to a High-Fat/Carbohydrate Meal Prevents Postprandial Endothelial Dysfunction and Oxidative Stress

Glucose fluctuations have been shown to alter endothelial cells by inducing markers of oxidative stress and DNA damage and the onset of a metabolic memory [141,142]. However, it appears that glucose fluctuations do not impact FMD shortly after intake (within 2 h) [143]. Beyond fluctuations in glucose concentrations, evidence has shown that it is the acute consumption of whole macronutrient meals that has the most influence on FMD within 6 h of intake [144].

Nuts have also been involved in improving endothelial function when combined with a meal. In healthy overweight or obese men, the acute consumption of a control shake significantly reduced FMD whereas a peanut shake, matched for nutrient content, did not significantly decrease FMD 4 h after the meal, regardless of the patients’ baseline cholesterol concentrations (total cholesterol -TC or low density lipoprotein-LDL) [139]. The peanut shake reduced the triglycerides area under the curve (TG AUC) by 32%. The impact of nuts on postprandial lipemia still needs to be clarified, as the results regarding improvements to postprandial VLDL, HDL, cholesterol efflux [145], and TG [139] are not always consistent [146].

There is some evidence that consuming walnuts improves postprandial endothelial function after a meal challenge in overweight or obese and hypercholesterolemic populations [135,136,147]. When measured with FMD, endothelial function improved over baseline by 64% following daily consumption for four weeks [147] or 24% after acute consumption [135]. In normocholesterolemic [135] or moderately hypercholesterolemic [136] populations only, a walnut meal has been shown to prevent postprandial endothelial dysfunction as assessed using both FMD and RHI measurements.

To determine the walnut component to which the effect on endothelial function could be ascribed, Berryman et al. [136] studied the effects of separated nut skins, de-fatted nutmeat, and nut oil derived from 85 g of whole walnut in mildly hypercholesterolemic individuals. The effect of walnut oil on fRHI differed from those of the skin and whole nut, and this might be related to its fatty acid composition. This is in line with the results of a study that compared two types of walnuts which differed in terms of their polyunsaturated fatty acid contents [148]. Finally, when compared with olive oil, which is quite low in polyunsaturated fatty acids (PUFA), the acute consumption of walnut with a high-fat meal improved endothelial function [135]. Taken together, these findings suggest a beneficial effect of plant PUFA, or in fact α-linolenic acid (ALA), on endothelial function.

Nuts have favorable effects on certain inflammation and oxidative status indices [149]. English walnuts contain the highest antioxidant content [150], and in healthy young adults the acute consumption of a walnut meal increased postprandial γ-tocopherol, catechins, and hydrophilic and lipophilic oxygen radical absorbance capacity (ORAC, a measure of the antioxidant capacity), while decreasing some markers of oxidative stress, such as MDA, when compared with a refined meal matched for energy nutrients [151]. These results suggest that walnuts exert antioxidant activities in both the lipid and aqueous plasma fractions. However, when comparing the antioxidant capacity of plasma regarding different walnut components in individuals with mild hypercholesterolemia, this antioxidant capacity (as assessed by the ferric reducing antioxidant potential, FRAP) was higher after the intake of walnut oil and skin compared with intake of the nutmeat [136].

Phenolic antioxidants may be more effective in MUFA-rich nuts, such as almonds and pistachios, than in PUFA-rich nuts [152]. One study reported that, in healthy individuals, the acute intake of almonds induced less protein damage during the postprandial period than parboiled rice/mashed potato, cheese, and butter meals, whereas the total antioxidant capacity did not differ between the groups [153].

As for the effects of pistachios on inflammation and oxidative stress, data are scarce in the acute setting. Nonetheless, several studies have shown chronic effects on various markers of oxidative stress in individuals with metabolic syndrome [154], hypercholesterolemia [155], and prediabetes [156], or in healthy populations [157,158]. By contrast, in obese people with metabolic syndrome, the acute consumption of pistachio meals had no significant postprandial effect on RHI [159]. It is still difficult to interpret the overall effects of pistachio nuts on postprandial inflammation and oxidative stress based on the results for various markers in isolation because many antioxidant components have been studied in plasma and tissues and there are few data to infer their final possible combined action. However, one study found a significant increase in blood antioxidant potential and lowering of MDA concentration (an indicator of lipid peroxidation) after substituting pistachio nuts for 20% of daily caloric intake for three weeks in a healthy population [158].

A review concluded that pistachios are singularly rich in nutrients and substances that exert antioxidant and anti-inflammatory effects that may be beneficial to cardiovascular health. There is evidence that three key nutrients/phytochemicals in pistachios could mediate these effects: carotenoids, ɣ-Tocopherol, and phenolic compounds [152].

### 5.2. Adding Fruit to a High Fat/Carbohydrate Meal Prevents Postprandial Endothelial Dysfunction and Oxidative Stress

The protective effects of extra virgin olive oil on postprandial oxidative stress have frequently been described during the past decade [160,161,162] and these effects appear to be comparable to those reported with walnuts. Indeed, the acute consumption of walnuts and olive oil in a high-fat meal by patients with hypercholesterolemia caused similar reductions in postprandial plasma concentrations of soluble inflammatory cytokines, adhesion molecules, and oxidized low-density lipoproteins. Only E-selectin levels fell more after the walnut meal than the olive oil meal. The authors concluded that both walnuts and olive oil preserve the protective phenotype of endothelial cells [31].

As are olives, avocados are a fruit that is specifically rich in MUFA (oleic acid) and n-6 PUFA (linoleic acid), and in this respect have also been studied recently in terms of their potential postprandial metabolic and vascular impacts. In overweight/obese individuals with elevated fasting glucose and insulin, the partial substitution of meal carbohydrates with avocado increased postprandial FMD [163]. However, the control breakfast did not result in a significant reduction in postprandial endothelial function as might have been expected. However, this result is important on practical grounds because the introduction of avocado in the meal represented only ~15% of the meal energy. The effect on FMD might, in part, have resulted from the effect on postprandial lipoprotein profiles, such as lower post-meal VLDL with avocado, which could be ascribed to the exchange of carbohydrates for MUFA. The avocado meal also caused a smaller increase in postprandial plasma insulin [163].

Because of their particular composition of nutrients and other substances, berries have also been studied in terms of their benefits on cardiovascular health [164]. In a well-designed study, Alqurashi et al. showed that in healthy overweight males in an acute setting, an acai-based shake (vs. a control shake) consumed alongside a high-fat breakfast significantly improved postprandial FMD [165]. Acai is well known for its high flavonoids content; however, the mechanism underlying the reported benefits of Acai still needs to be elucidated and further research is required to understand the degree to which this effect could be extended to other berries. Additional positive findings have been reported for other berries such as blueberry and raspberry, and possible mediation by polyphenols has been considered. In healthy males, the acute consumption of processed or unprocessed blueberries caused changes to the profile of polyphenols but not the amount, resulting in different patterns of increase in polyphenol metabolites in the plasma but similar improvements in postprandial FMD [166]. After the consumption of raspberries, increases in plasma urolithin metabolites were found to be associated with improvements to endothelial function [167]. Interestingly, plasma total nitrite concentrations have been reported to rise significantly during the 2 h following intake of cranberries, suggesting that polyphenols increase postprandial circulating nitric oxide and mediate the maintenance of postprandial endothelial function [168].

Berries are rich in phytochemicals, and particularly phenolic compounds (2/3 flavonoids such as anthocyanins, catechins, quercetin, and kaempferol, and 1/3 phenolic acids such as ellagic acid), which are considered to be potent antioxidants inasmuch as they are able to scavenge ROS, chelate metal ions in vitro, and act synergistically between themselves and with micronutrients such as ascorbate and tocopherol [165,169,170]. The effects of berries on post-meal oxidative stress have been described in both the acute [171,172] and chronic settings [172]. In a chronic context, berries may exert anti-oxidative and anti-inflammatory effects by modulating mRNA expression in overweight and hypercholesterolemic individuals. Indeed, a study showed that the intake of an aqueous extract of wolfberry fruit (goji) once a day after a meal for eight weeks significantly decreased erythrocyte superoxide dismutase activity, DNA damage in lymphocytes, and the expression of TNF, IL-6, and other mRNAs related to oxidative or inflammatory stress. In addition, superoxide dismutase (SOD) expression in whole-cell extracts was down-regulated [173].

The effects of strawberries on postprandial hyperlipidemia and oxidized low-density lipoprotein cholesterol (LDL) have previously been studied in hyperlipidemic and overweight individuals using a control beverage supplemented with strawberry powder at a dietary dose (equivalent to 110 g fresh strawberries) or a placebo beverage (matched for energy, macronutrient, micronutrient, and fiber contents) given with a high-fat test meal. In the acute setting, the strawberry beverage (vs. the control) lowered postprandial increases in TG, HDL, and OxLDL at 3, 4, and 6 h after the meal. In the chronic setting, after a 6-week period, the strawberry beverage lowered mean cholesterol, LDL, TG, and OxLDL concentrations (when adjusted for fasting values) following the intake of a high-fat meal [172].

Berries reduce the lowering of ORAC that is usually reported after carbohydrate meals. Furthermore, in healthy women, when adding grape and blueberry powder to a carbohydrate meal, ORAC increases within 2 h of intake. When comparing the AUC for the change in plasma hydrophilic ORAC-FL over 4–5 h after a meal, Burton-Freeman et al. found that the decrease was halved after a grape and blueberry supplemented meal as compared to the control meal [171]. The ultimate health impacts of such postprandial changes to ORAC still need to be determined.

Alternatively, in adults with type 2 diabetes, the addition of cranberries (40 g dried) to a high-fat fast-food-style breakfast lowered some biomarkers of inflammation and lipid oxidation, such as serum IL-18 and MDA, 4 h after the meal, although no significant differences in postprandial concentrations of CRP and IL-6 were observed [168]. Postprandially, a meal composed of an antioxidant-rich concentrate of berry added to a turkey burger and in the water consumed during the meal blunted the postprandial increase in MDA, decreased protein carbonyls (a marker of oxidative stress on protein), and increased plasma antioxidant activity [174].

A similar series of protective effects on postprandial inflammation in mononuclear cells has also been reported regarding the consumption of orange juice with a high-fat meal [104]. Consuming orange juice (300 kcal, i.e., ~600 mL, versus water or a glucose solution) with the meal, lowered the postprandial production of ROS by blood polymorphonuclear cells and resulted in less activation of inflammatory pathways such as mitogen-activated protein kinase (MAPK) and suppressor of cytokine signaling 3 (SOCS-3) in mononuclear cells. As with the aforementioned study, orange juice also lessened postprandial low-grade endotoxemia and the expression of toll-like receptor 4 (TLR-4) [104].

Regarding oxidative stress and the effects of fruit juice, the results should be interpreted with caution as most studies assessing the effects of fruit-based beverages on postprandial stress used as a control a drink matched for macro- and micronutrients and not simple water. Therefore, what was being tested was not the fruit-based juice itself but rather the phytochemicals it contained in the context of a drink and a high-fat meal [175].

By contrast, acute avocado consumption was not associated with postprandial changes to biomarkers of inflammation or oxidative stress/damage to MCP-1, tumor necrosis factor alpha (TNF-α), or Ox-LDL [163].

## 6. Key Phytochemicals Identified as Mediating Postprandial Antioxidant and Anti-Inflammatory Effects

According to the same type of study design, it has been reported that red wine (but not vodka) consumed with a high-fat meal prevented the postprandial activation of NF-κB in mononuclear cells [176]. Indeed, the ingestion of wine with a meal has been reported to reduce postprandial oxidative stress, although the markers chosen for most studies were of limited value [177]. It has also been shown that the consumption of other foods and nutrients does not result in the postprandial inflammation and dysfunction that are induced by high saturated fat and high sucrose loads.

In the context of elucidating the complex effects of wine on endothelial function and postprandial inflammation, it was reported that combining muscadine grape polyphenols with resveratrol—a phenolic compound in red wine that has been long largely studied for various anti-inflammatory effects [178]—reduced postprandial increases in a set of pro-inflammatory and inflammatory markers in mononuclear cells, such as the expression of IL1-β and SOCS-3 [179]. Because the dose of resveratrol (100 mg) used in this study was very high when compared to the amounts found in wine [180,181,182,183], the results cannot be used to conclude that wine polyphenols and resveratrol are candidates for a potentially favorable effect of wine on postprandial inflammation [184,185]. However, they offer a good example of the potential effects of combining different chemicals at neutraceutical doses on postprandial dysfunctions.

The effects of the resveratrol and polyphenols combination were considered in detail in the same study, and this work also provided some interesting insights into the possible mechanisms underlying prevention of the initiation of inflammation in mononuclear cells in the postprandial setting. The combination of resveratrol and polyphenols largely reduced the increase in the expression of the p47 NADPH subunit, which is known to be associated with a postprandial increase in oxidative stress in mononuclear cells. Furthermore, the supplement increased the binding activity of Nrf-2 and the expression of some target genes. Because Nrf-2 is a transcription factor that mediates the physiological antioxidant response to oxidative stress, this supplementation may have limited the production of ROS yet evoked a higher protective antioxidant response, and the nrf-2 pathway might be important in mediating the adverse effect of triglyceride-rich lipoprotein on vascular health [186]. However, in our view, because of the delay required for this antioxidant response to take effect, it might not generally account for the series of protective effects that appear acutely in the postprandial phase. Other authors have confirmed that grape powder (in quantities compatible with dietary modulation) increases the expression of Nrf-2 acutely during a high fat carbohydrate meal [187]. Another result of importance to our understanding of the pathogenesis of postprandial adverse effects and that of the effect of the supplement in the study by Ghanim et al. is that the supplement also reduced or prevented postprandial low-grade endotoxemia, plasma lipoprotein binding protein, and TLR-4 expression in mononuclear cells. As discussed above, an increase in the translocation of endotoxins from the gut has been proposed as a mechanism for the adverse postprandial effect of high-fat meals on low-grade inflammation and endothelial dysfunction [75,188,189]. However, such a mechanism may not strictly require TLR-4 mediation, but rather may act through a combined interplay between TLRs [190]. Therefore, the protective effect of the supplement may be mediated, at least in part, by a reduction in postprandial low-grade endotoxemia and downstream pro-inflammatory signaling [179], although the potential underlying mechanisms for an acute reduction in endotoxemia still need to be fully elucidated.

With respect to berries, urolithins and ellagic acid appear to be the best candidates for their anti-atherogenic effects. In quantities compatible with dietary modulation, these phytochemicals have displayed their potential to affect key processes in the development and progression of atherosclerosis in vitro, such as endothelial activation and resulting monocyte recruitment, cholesterol transport, and foam cell formation [191].

Some polyphenolic compounds are attractive candidates to explain the effects of orange juice. In this regard, it was shown that the consumption for four weeks of 500 mL orange juice, or hesperidin (the major flavonoid in orange juice), increased microvascular endothelium-dependent function during the peak of hesperidin absorption [192]. This result cannot directly be extrapolated to the postprandial phase. However, because hesperidin has a short half-life in plasma, its effect may be mostly transient so it may operate acutely during the postprandial period, with favorable effects on macro- or micro-vascular endothelial function or other related pro-inflammatory postprandial features. More recently, in adults with hypertriglyceridemia or who were overweight/obese and subjected to a double high-fat meal challenge, a study reported that various orange-based drinks containing flavanone (vs. an isoenergetic control) alleviated the postprandial decrease in FMD 7 h after a high-fat meal. The effects were similar despite variations by a factor of four in the amount of flavanone in the drinks. However, the effect on FMD at 7 h coincided with the peak of naringenin and hesperidin metabolites being found in the plasma, and the fraction of hesperidin metabolites assayed in plasma predicted, in part, the magnitude of the changes to FMD [193]. Salden et al. did not evidence any effect of supplementation with hesperidin 2S in their study sample as a whole, either with acute postprandial testing, or after six weeks of supplementation. In individuals with a normal or high baseline FMD (60% of the total sample), hesperidin 2S improved FMD and reduced adhesion molecules after a HFM, when the latter was given after six weeks of supplementation [194].

It is difficult to draw any firm conclusions from the literature on the postprandial effects of polyphenols. First, many studies have investigated the effects of polyphenol-rich foods (such as cocoa, grape, or berries) or food preparations (e.g., juices) rather than purified and well-characterized extracts. Again, adding a food/ingredient with a significant mass and energy (e.g., 500 mL juice) to a challenge meal tends to affect the kinetics of postprandial metabolism directly, and the results are therefore difficult to analyze. More recently, studies have used ingredients such as powders, and control treatments matched for macronutrient content, which is more useful when trying to ascribe the effects to polyphenolic fractions [172,187,195]. Second, studies have resorted to different postprandial endpoints and markers, in limited numbers, giving rise to highly fragmented findings. For instance, the consumption of a juice rich in blackcurrant polyphenols (as compared to a well-made placebo drink) was shown to improve postprandial oxidative status, but in vivo evidence for postprandial anti-inflammatory effects was lacking [196] as was evidence for a beneficial effect on vascular reactivity [197]. Likewise, an anthocyanin-rich blackcurrant extract lowered postprandial glucose and insulin after a high-carbohydrate meal but did not affect 8-isoprostane F2α (a stable and reliable marker of overall lipid peroxidation [164]) or arterial stiffness; endothelial function was not measured [198].

Many phenolic compounds have shown that they can reduce postprandial oxidative stress or acutely affect parameters for oxidative stress [164,199]. This has been largely documented for cocoa flavanols and grape polyphenols [200]. Potential mechanisms of action have also been reported. In vitro, a grape seed extract and a strawberry powder activated NO synthesis pathways in endothelial cells [201,202]. Cocoa flavanols acutely increased plasma concentrations of nitroso compounds, reduced arginase activity [203,204], affected pro-inflammatory pathways in vitro [205], and acutely improved endothelial dysfunction [206,207,208]. However, for many polyphenolic compounds with interesting in vivo effects after ingestion, the evidence remains limited regarding their effects on important endpoints of cardiometabolic health, such as endothelial function in humans [196,209].

## 7. Conclusions

As we have shown in this review, acute supplementation with certain whole foods, ingredients, nutrients, and phytochemicals can prevent postprandial endothelial dysfunction and inflammation. Because many studies have lent credence to the current paradigm that oxidative stress mediates adverse postprandial effects [58], further efforts are necessary to determine whether nutrients and substances that display postprandial antioxidant effects also reduce postprandial low-grade inflammation and vascular endothelial dysfunction. Future studies also need to investigate other mechanisms that are good candidates for the acute effects of nutrients during a high-fat meal, such the induction of low-grade endotoxemia. These studies could take advantage of simultaneously analyzing the effects on different endpoints and using various markers. It would be interesting to further clarify the degree to which certain nutrients (e.g., some amino acids) and other substances (in particular various polyphenols) affect potential underlying mechanisms that are directly or indirectly related to oxidative stress, including NO and nitroso-compound metabolism, induction of the antioxidant defense system, the delicate redox status in tissues, and insulin-related signaling pathways [203,210,211,212,213]. Further studies could also profitably investigate acute variations in the metabolism of arginine and related compounds (such as homoarginine and methylated arginine) during the postprandial period, and their potential modulation by the nature of the meal [214,215].

The metabolic utilization and effects of any nutrients added to a high-fat high sucrose meal are basically postprandial (e.g., amino acids). Many other dietary substances also exhibit an acute postprandial metabolism; in particular, despite huge heterogeneity, many polyphenols (e.g., flavonoids) and their metabolites display early plasma peaks (e.g., at 2 h) and a short half-life in plasma after ingestion [216]. We can therefore expect that many nutrients and other substances exert most of their biological effects during the postprandial period. This factor warrants dedicated investigations of their specific effects under adverse postprandial conditions.

Finally, we can conclude that based on a very large set of data, the present paradigm is that the postprandial occurrence of cardiometabolic-related dysfunctions, including postprandial inflammation and endothelial dysfunction, are pathogenic to the initiation and progression of MS. The high-saturated-fat/high-sucrose model is therefore highly relevant to preventive nutrition, and as it is also practical for the conduct of human trials, it can be used to study the benefit of nutrients and other dietary substances when added to a challenge meal or consumed immediately beforehand. Although many studies have addressed the postprandial effects of nutrients and other substances, the literature remains largely fragmented. In particular, some nutrients and substances have been shown to lower postprandial oxidative stress and impact inflammatory-related pathways, but further studies are needed and should involve final critical endpoints such as endothelial dysfunction. Nevertheless, we found that nuts, l-arginine, polyphenols from berries, and citrus are good candidates for acute and multiple protective effects during the postprandial phase, and the data so far warrant further investigations involving multiple clear endpoints and valid, sensitive markers to ascertain the global picture.
